# Slip-Effect Functional Air Filter for Efficient Purification of PM_2.5_

**DOI:** 10.1038/srep35472

**Published:** 2016-10-17

**Authors:** Xinglei Zhao, Shan Wang, Xia Yin, Jianyong Yu, Bin Ding

**Affiliations:** 1 Key Laboratory of Textile Science & Technology, Ministry of Education, College of Textiles, Donghua University, Shanghai 201620, China; 2 Nanofibers Research Center, Modern Textile Institute, Donghua University, Shanghai 200051, China

## Abstract

Fabrication of air filtration materials (AFM) that allow air to easily flow through while retaining particles is a significant and urgent need due to the harmful airborne particulate matter pollution; however, this is still a challenging research area. Herein, we report novel slip-effect functional nanofibrous membranes with decreased air resistance (reduction rate of 40%) due to the slip flow of air molecules on the periphery of nanofibers. This was achieved through careful control over the diameters of electrospun polyacrylonitrile fibers and aperture size of fiber assembly. Fiber assembly with 86% of fiber diameters between 60–100 nm was found to be most effective for slip flow, as these diameters are close to the mean free path of air molecules (65.3 nm). Significantly, an equilibrium factor *τ* = *d*_*f*_/*d*^2^ has been introduced to elucidate the effect of distance of adjacent fibers on the drag force of airflow. Furthermore, the most effective aperture size (>3.5 μm) for slip-effect has been determined. Ultimately, the new material displayed low air resistance of 29.5 Pa, high purification efficiency of 99.09%, good transmittance of 77%, and long service life. The successful fabrication of such materials can facilitate the development of high-performance AFMs for various applications.

Fine particulate matters (PM) are severe pollutants characterized by their small size, large specific area, and easy adherence to poisonous substances, hence, they constitute a serious threat to public health^1^,^2^,^3^. Reports from the International Agency for Research on Cancer have shown that PM_2.5_ (with particle sizes ≤2.5 μm) can cause congestive heart-failure, coronary artery blockage and lung carcinoma^4^, all of which have the potential to result in death. A recent report from the United States Environmental Protection Agency has shown that 2.1 million deaths are incurred every year due to the rising concentrations of PM_2.5_ worldwide^5^. Therefore, to eliminate PM_2.5_ from polluted air, various technologies including electrostatic precipitation^6^,^7^,^8^ and electrostatic enhanced fiber filtering^9^, have been exploited. Both of these techniques can effectively adsorb particles due to fortissimo electrostatic force. Unfortunately, poor safety, high energy consumption, and short service life have severely restricted the application of these techniques^10^,^11^.

Fiber-based air filters have become a very feasible, efficient and promising technology to combat air pollution due to their reticular support structure and tortuous pore channels, which allow the effective passage of air molecules, while trapping the particulate matter^12^,^13^. For this reason, various fiber-fabrication technologies have been developed to prepare high-performance fibrous materials for air filtration, such as melt-blown^14^,^15^, needle-punched^16^,^17^, and papermaking methods^18^,^19^. Nevertheless, conventional fibrous materials prepared by the above mentioned methods suffer from high air resistance caused by the direct impact of air molecules with the fibers, which results in the transition of airflow state due to the large diameter of fibers and dense structure of fiber assembly^20^,^21^. Moreover, these fibrous materials also face the problem of low filtration efficiency due to large pore size^22^. Electret materials used for air filtration have the advantage of low initial pressure drop, but they suffer from easily dampened electret effect and safety hazards.

Electrospinning is an effective fiber fabrication technique that can overcome the drawbacks of conventional methods, because it can provide fibrous materials having ultrathin diameters (10–1000 nm), extensively interconnected pores, and adjustable porosity^23^,^24^,^25^. More importantly, aerodynamic behavior of airflow around the periphery of fibers would be changed dramatically when the diameter of fibers is reduced to nanoscale^26^. The air flow is correlated with the mean free path (*λ*) of air molecules, and can be described as^27^,^28^: 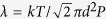, where *k* represents the Boltzmann constant; *d* signifies the effective diameter of air molecules; *T* is the temperature of air; and *P* is the standard atmospheric pressure. Generally, there are 2.54 × 10^7^ air molecules in a cube having sides of one micron length. Two adjacent air molecules can be separated by a mean spatial distance of 0.0034 μm when air is at standard temperature (*T* = 288 K) and atmospheric pressure (*P* = 1.01 × 10^5^ N m^−2^), and the mean free path of air molecules (*λ*) is obtained as 65.3 nm. Interestingly, when the fiber diameter is close to 65.3 nm, the velocity of airflow is non-zero on the surface of single fiber due to the slip flow phenomenon, which will result in significant reduction of drag force of the airstream^29^.

Combining the mean free path of air molecules and diameter of nanofibers, the flow regime of the gas can be divided into four types^27^: free molecular flow regime, transition flow regime, slip flow regime, and continuum flow regime. The different flow regimes can be defined by the Knudsen number^26^: *Kn* = 2*λ*/*d*_*f*_. The diameter of electrospun nanofibers is appropriate for the transition flow regime and the corresponding value of Kn is between 0.25 to 10. Up to now, only a few studies have investigated the slip-flow effect of electrospun nanofibrous membranes. In one such study, Hung *et al*. fabricated Nylon 6 nanofibers with diameters between 180 to 94 nm to investigate the filtration of nano-aerosols with low Peclet number, but unfortunately the reduction of fiber diameter resulted in steadily increasing pressure drop^30^. Also, Balgis *et al*. discovered that a gentle increase in pressure drop along with improved filtration efficiency could be obtained using polyacrylonitrile nanofibers with diameters of 36.5–300 nm^31^. However, both of the above studies have several drawbacks: (1) the optimal range of fiber diameter was not defined, (2) the relationship between pore structure and slip effect was not elucidated, resulting in high air resistance. Accordingly, the two major challenges that need to be addressed are fabricating slip-effect functional fibrous materials with optimum fiber diameter and establishing the relationship between membrane structure and slip-effect to determine the effective pore size.

In this study, we present a facile strategy to fabricate slip-effect functional PAN fibrous membranes with decreased air resistance by regulating the diameter of single fibers and pore size of fiber assembly. The contribution of slip-effect to the reduction of air resistance with the decrease of fiber diameter was thoroughly investigated. Also, the airflow around single fibers and inside the fiber assembly was simulated for an in-depth investigation of the airflow feature based on bulk statistical properties. Notably, an equilibrium factor *τ* = *d*_*f*_/*d*^2^ was introduced to uncover the mechanism of the reduction of air resistance. Then, the optimal range of pore size (>3.5 μm) for the slip-effect was determined. Finally, the PAN fibrous membrane was prepared with these optimized features and thoroughly tested for filtration efficiency, air resistance and durability. This work is expected to provide an efficient approach to designing improved filtration materials.

## Results

### Fabrication of slip-effect functional fibrous AFMs

The objective of this paper is to fabricate slip-effect functional nanofibrous membranes for air filtration with reduced air resistance and improved filtration efficiency for effective purification of PM_2.5_ in air. To achieve this target, we designed the fibrous AFMs based on the following three criteria: (1) the nanofiber-based AFMs must consist of uniformly distributed and bead-free nanofibers; (2) the diameter of nanofibers should be similar to the mean free path length of air molecules, which would help air molecules to bypass the nanofibers with maximum probability; (3) the nanofiber-based AFMs should have optimal aperture size to avoid weakening or elimination of the slip-effect induced by the interaction of airflow stream around the periphery of adjacent fibers. The first requirement was achieved by the versatile and efficient electrospinning fiber-fabrication technology, which involved carefully selecting the appropriate molecular weight of polymer and properly regulating conductivity of polymer solution, both of which allow control of the fiber diameter.

**Figure 1:**
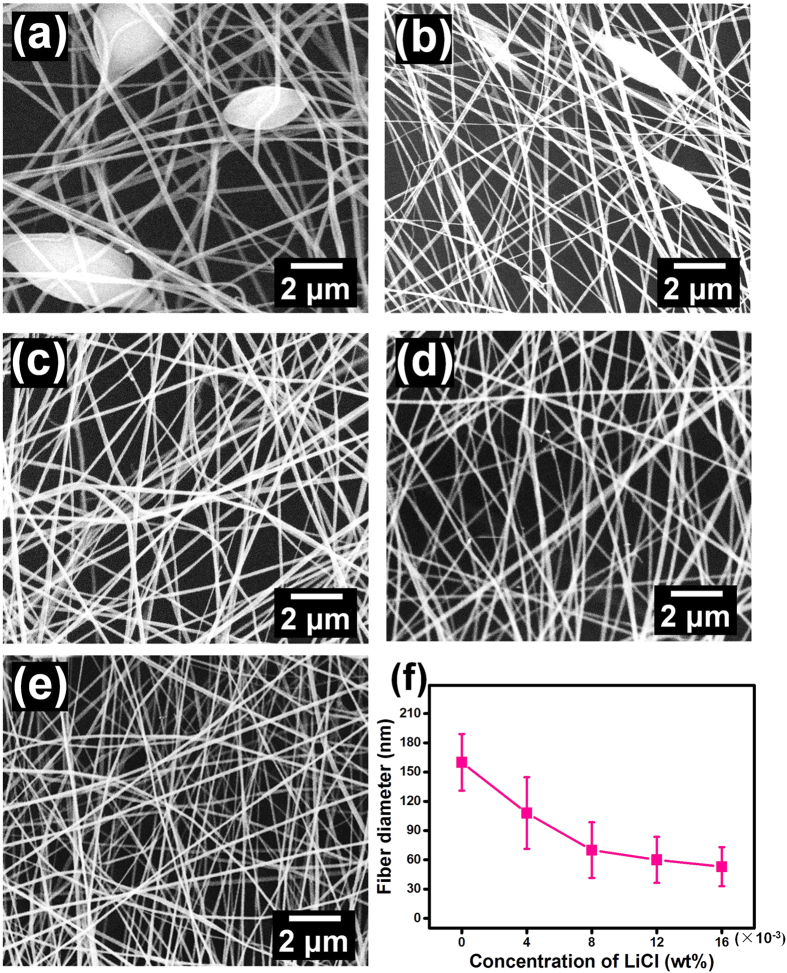
Morphology and fiber diameter of PAN fibrous membranes. SEM images of PAN fibrous membranes fabricated from polymer solutions with various concentrations of LiCl: (**a**) 0, (**b**) 0.004, (**c**) 0.008, (**d**) 0.012, and (**e**) 0.016 wt%, respectively. (**f**) Fiber diameter of corresponding PAN fibrous membranes.

To obtain the fibers with diameter in the vicinity of mean free path of air molecules, polyacrylonitrile (PAN) with low molecular weight was selected to fabricate slip-effect functional nanofibers. Lithium chloride (LiCl) was used to enhance the solution conductivity to obtain the bead-free nanofibers. As shown in Fig. 1a, the pristine PAN fibers displayed bead-on-string binary structures, comprised of slender fibers with diameters between 70–230 nm and microsized spindle shaped beads with an average size of 1.71 μm along the fiber length direction (Supplementary Fig. S1). The formation of this unique structure could be due to the use of a low viscosity polymer solution (Supplementary Fig. S2), which could disturb the balance between viscoelastic force, surface tension and electrostatic repulsion, inducing instability of the Taylor cone during electrospinning^32^. Interestingly, inclusion of 0.004 wt% LiCl reduced the fiber diameter, resulting in 70% of the fibers in the range of 80–120 nm and smaller bead size of 0.98 μm (Fig. 1b and Supplementary Fig. S1). These effects could be due to the increased solution conductivity by LiCl^33^ (Supplementary Fig. S2). However, further increase in the concentration of LiCl to 0.008 wt% resulted in more uniform distribution of fibers with 86% of fiber diameters between 60–100 nm and the disappearance of bead structures (Fig. 1c and Supplementary Fig. S1). Importantly, the average diameter of fibers further decreased with continuous increase in the concentration of LiCl, achieving the lowest value of 53 nm for PAN-16 (Fig. 1d–f and Supplementary Fig. S1), which is close to the limit for electrospun fiber diameter. This low value of fiber diameter indicated that the obtained fibrous membranes could be suitable for exploration of the slip-effect. The SEM images showing a large area of the PAN fibrous membranes fabricated from polymer solutions containing various LiCl concentrations of 0.004, 0.008, 0.012, and 0.016 wt% are presented in Supplementary Fig. S3.

### Experimental verification of slip-effect

**Figure 2:**
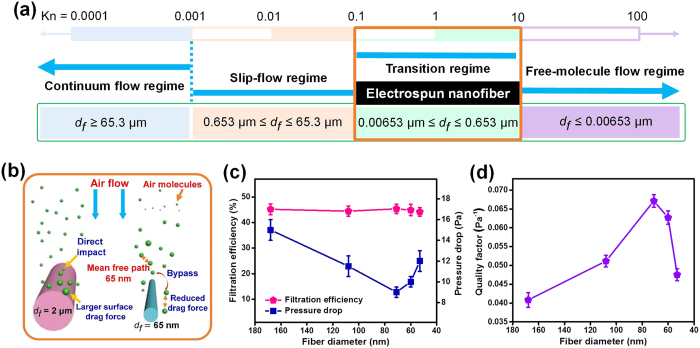
Knudsen number regimes, mechanism of slip flow and filtration properties. (**a**) The schematic representation of the scale bars of flow regime. (**b**) Schematic showing the mechanism of slip flow. (**c**) The relationship between filtration performance and fiber diameter. (**d**) The relationship between QF value and fiber diameter.

The appearance of the slip-effect is determined by the flow regime around the periphery of single fibers, which could be controlled by the diameter of fibers at standard atmospheric condition. Figure 2a shows scale bars of all the relevant sizes within the four flow regimes mentioned earlier. Together with the fiber diameter of PAN fibrous membranes, which was in the vicinity of the mean free path of air molecules, the air flow around fibers belonged to transition flow regime. The corresponding *Kn* values of PAN-0, PAN-4, PAN-8, PAN-12, and PAN-16 were 0.39, 0.60, 0.92, 1.08, and 1.23, respectively. In contrast to the impact behavior of air molecules with fibers in continuum flow regime, segmental slip flow of air molecules over the fiber surface would take place and become more significant with the reduction of fiber diameter in the slip-flow regime. It is worth noting that there was also direct impact of air molecules with fibers in the slip-flow regime, causing larger drag force on the periphery of single fibers compared to the transition regime, as shown in Fig. 2b. In the transition regime, the air molecules bypassed around the fibers and the drag force of airstream also decreased. Based on this, we could reasonably assume that the PAN fibrous membranes possessed the slip-effect feature, which could lower the air resistance.

The resistance of PAN fibrous membranes to the transmission of air molecules was measured to verify the slip flow of air molecules on the surface of the nanofibers. The airflow resistance can be measured by the pressure drop in the fiber assembly and indicate the presence of the slip-effect. More importantly, the short electrospinning time resulted in fewer fibers, and less changes in the thickness and porosity (Supplementary Fig. S4). Figure 2c shows the plots of transformation curves of pressure drop in PAN fibrous membranes with reduced fiber diameter while maintaining the filtration efficiency of membranes at consistent average level (44~45%). Three distinct variation regimes could be clearly seen: a linear rapidly descending region with the average fiber diameter *d*_*f*_ changing from 168 to 71 nm, a slowly rising region with the average fiber diameter *d*_*f*_ decreasing from 71 to 60 nm, and a rapidly rising region with further reduction of average fiber diameter from 60 to 53 nm. The reduction of pressure drop from 15 to 9 Pa (40% reduction) in the rapidly descending region indicated that the air molecules could more easily travel through the membranes as the fiber diameter gradually approached the mean free path of air molecules due to the developing slip flow^34^. Interestingly, the resistance of fibers to air molecules showed an increase of 11% (from 9 to 10 Pa) in the slowly rising region, which was lower than the 20% increase (from 10 to 12 Pa) in the rapidly rising region, indicating that the slip-effect was gradually weakening with the reduction of fiber diameter. The weakening of the slip effect could be due to the mutual interaction between airflow around nearby fibers^35^. This interesting phenomenon was contradictory with the Knudsen theory which states that the pressure drop decreases with the reduction of fiber diameter^36^.

In addition, the quality factor (*QF*) was used to evaluate the filtration performance of the obtained membranes. This factor can be defined as: *QF* = *−ln(1 − η)/Δp*, where *Δp* and *η* represent pressure drop and filtration efficiency^37^. As illustrated in Fig. 2d, due to the slip-effect, the PAN-8 membrane showed a higher *QF* value of 0.067 Pa^−1^ than the other membranes, confirming that the slip-effect is responsible for the reduction of pressure drop.

### Simulation of slip-effect

**Figure 3:**
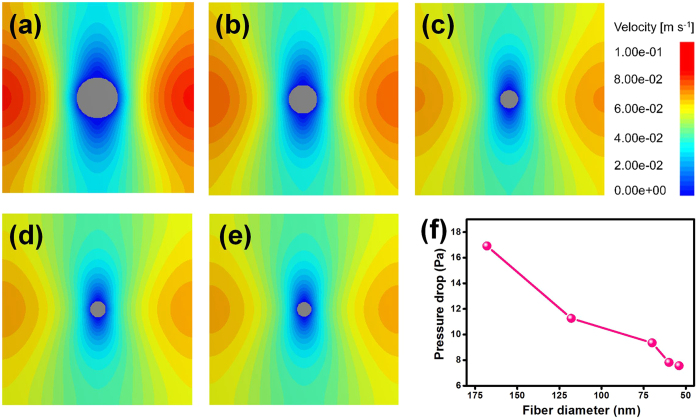
Airflow distribution around the periphery of fibers and simulated pressure drop of single fibers. The airflow state around the cross-sections of the relevant single fiber with various diameter: (**a**) 168, (**b**) 108, (**c**) 71, (**d**) 60, and (**e**) 53 nm. (**f**) Simulated pressure drop of relevant single fiber at 5.3 cm s^−1^.

In order to study the distribution of airflow field on the periphery of single fibers, computer simulation was performed based on the statistical average fiber diameter of PAN fibrous membranes *via* Geodict. Herein, Stokes flow was the prevailing air fluid, in which the Reynolds number was small (*Re* ≤ 1) and inertia force was far lower than viscous force^38^. Periodic boundary conditions were employed to maintain the simulation system constant and eliminate the influence of boundary effect. In addition, the flow rate and temperature of the simulated airstream were kept as 5.3 cm s^−1^ and 20 °C, respectively, to generate the most authentic test conditions. As shown in Fig. 3a–e, the airflow velocity on the periphery of nanofibers with various diameters exhibited a gradually increasing trend, which could be ascertained from the change in blue color within the streamline. This increase in velocity could be due to the reduction of viscous force induced by random motion and impact of air molecules with the fiber diameter being close to or less than the mean free path of air molecules^39^. In addition, as illustrated in Fig. 3f, due to the reduction of viscous force of air, the pressure drop of fibers between upper surface and bottom surface falls off substantially. This phenomenon is different from the results observed in the case of PAN fibrous membranes. This is because there is no mutual interaction between airflow around the nearby fibers in the case of single fibers (Supplementary Fig. S5). More importantly, this is consistent with the Knudsen theory that when fiber diameter is close to or less than the mean free path of air molecules, then the air molecules can bypass the fibers freely^26^,^34^.

**Figure 4:**
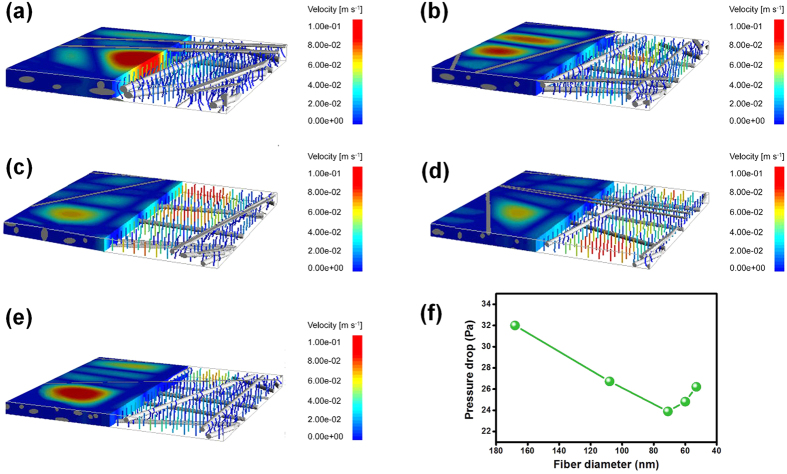
Airflow distribution inside the fibrous membranes and simulated pressure drop of fibrous membranes. The airflow state of relevant PAN fibrous membranes with various diameter: (**a**) 168, (**b**) 108, (**c**) 71, (**d**) 60, and (**e**) 53 nm. (**f**) Simulated pressure drop of relevant PAN fibrous membranes at 5.3 cm s^−1^.

In contrast to the simple structure of single fibers, the fiber assembly of the membranes consisted of an intricate hierarchical structure and complex-shaped aperture architecture, which could have significant effects on the airflow around the periphery of fibers^40^. Therefore, we performed computer simulation of the airflow inside the fiber assembly based on the weight, packing density, porous structure and relevant structural parameters of the as-spun PAN fibrous membranes to further clarify the distribution of airflow field and pressure drop of the fibrous membranes. Also, the simulation parameters were identical to those of the single fiber. As illustrated in Fig. 4a–c, the color of airflow streamline inside the membranes transformed from fully deep blue to half red, indicating that the ability of air molecules to bypass the nanofibers inside the membranes was enhanced as the average fiber diameter decreased. Moreover, the viscous force of the airstream gradually weakened, which was similar to the case of single fiber. Notably, the red streamline of airflow inside the membranes began to decrease and eventually completely vanished with further reduction of the average fiber diameter from 60 to 53 nm. Thus, the slip-effect of single fibers and viscous force of airstream are affected by the hierarchical structure and aperture architecture of fibrous membranes. In addition, as shown in Fig. 4f, plotting the curves of simulated pressure drop of PAN-0, PAN-4, PAN-8, PAN-12, and PAN-16 fibrous membranes versus their average fiber diameter also exhibited three distinct variation regions. This is consistent with the experimental results discussed earlier, thus confirming the contribution of the slip-effect to the reduced air resistance of fibrous membranes.

### The relationship between pore structure and slip-effect

To understand the mechanism of change in air resistance, we intensively investigated the relationship between pore structure and slip-effect based on the drag theory, which expounds that the pressure drop in fibrous materials with unit thickness is the sum of the whole drag force on the fibers^41^. Drag force on a unit length of fiber with diameter *d*_*f*_ transverse to the flow in a membrane is directly proportional to the drag coefficient, which can be defined by^35^:


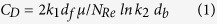


where *k*_1_ signifies shape factor of fiber, *k*_2_ represents a constant related to fibers arrangement, *d*_*f*_ is the diameter of fibers, *N*_*Re*_ = *ρvd*/*μ* is Reynolds number, *d*_*b*_ represents the distance between the adjacent fibers and *μ* is the viscosity coefficient of the fluids. This was based on the assumption that a membrane has the identical distance between fibers in all three directions. However, for nanofibrous membranes with oriented randomly structure, the distance of adjacent fibers is difficult to determine. Therefore, we introduced the equivalent diameter *d* of pores developed by random arrangement of fibers to represent the distance of adjacent fibers. Based on this, equilibrium factor *τ* = *d*_*f*_/*d*^2^ can be established to reveal the relationship between porous structures and drag force of airstream on the periphery of fibers. This would then indicate the contribution of slip-effect to the reduction of pressure drop in fibrous membranes.

**Figure 5:**
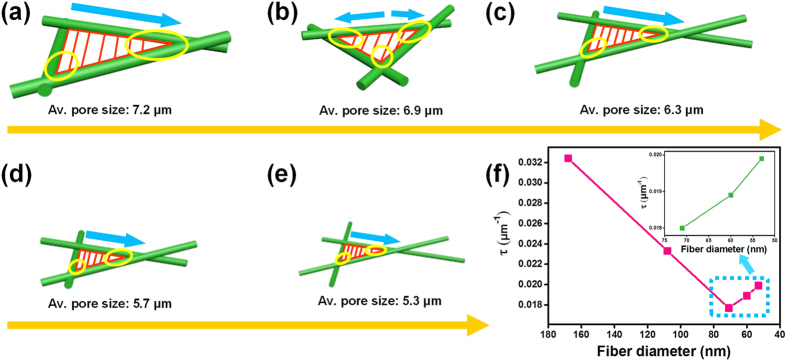
Mechanism of change in air resistance. Schematics showing the pore size transformation of PAN fibrous with different fiber diameter: (**a**) 168, (**b**) 108, (**c**) 71, (**d**) 60, and (**e**) 53 nm. (**f**) Equilibrium factor of PAN fibrous membranes with different fiber diameter.

As illustrated in Fig. 5a–e, decreased size of the triangular pore developed by the overlap of fibers could be clearly correlated with the reduction of fiber diameter, which resulted in significant decrease of interfibrous distance (red lines). It is worth noting that the minimum distance of adjacent fibers is reflected in the overlapping contact denoted by the yellow circles, which could have a large influence on the variation of slip-effect. The pore size distribution curves show that the pore size of PAN-0, PAN-4, PAN-8, PAN-12 and PAN-16 fibrous AFMs were in the range of 5–8 μm, with a large proportion of peaks concentrated at 7.2, 6.95, 6.33, 5.7, and 5.3 μm, respectively, (Supplementary Fig. S6). These pore sizes would greatly influence the flow around neighboring fibers. As illustrated in Fig. 5f, the curve of equilibrium factor *τ* exhibited two distinct regimes with the decrease of fiber diameter: a linear decrease stage (from 3.2 to 2.5) with *d*_*f*_ decrease from 168 to 71 nm, and a slow linear increase stage (from 1.9 to 1.99) with *d*_*f*_ decrease from 71 to 53 nm. This behavior was consistent with the variation of pressure drop, indicating that the behavior of airflow and drag force at the fibers’ periphery will be altered when two neighboring fibers come closer.

**Figure 6:**
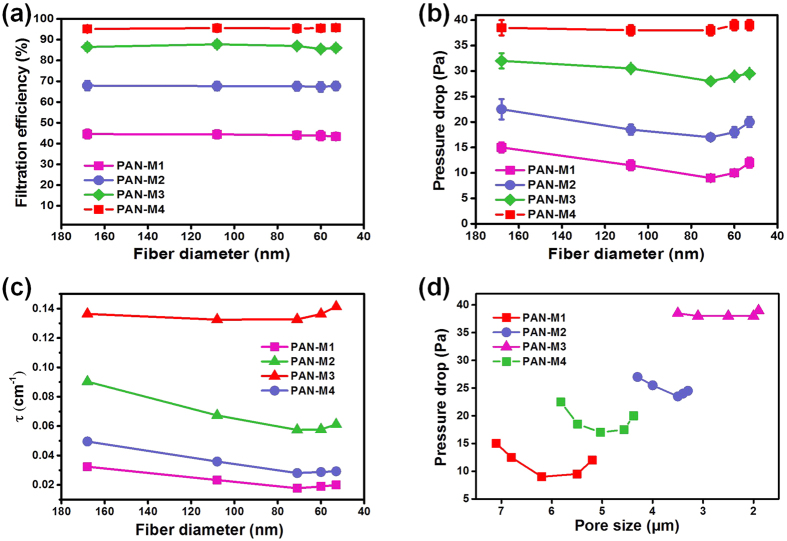
Filtration properties, equilibrium factor, and the optimal pore size of the PAN fibrous membranes with various thickness. (**a**) Filtration efficiency, (**b**) Pressure drop, and (**c**) Equilibrium factor of PAN fibrous membranes with various levels of filtration efficiencies fabricated from various concentrations of LiCl. (**d**) Structure-function relationship between pressure drop and pore size.

To further investigate the scaling law of the slip-effect change with gradually decreased pore size and to determine the optimal size of pores for the maximum slip-effect, we fabricated several PAN fibrous membranes with nearly identical filtration efficiencies by controlling the electrospinning time, as illustrated in Fig. 6a. Interestingly, the PAN membranes with average filtration efficiency of 66–68% and 86–88% first exhibited a distinct decrease of pressure drop with the reduction of fiber diameter from 168 to 71 nm and subsequently showed an increasing trend (as shown in Fig. 6b). This behavior was similar to that of PAN membranes with filtration efficiency of 44–45%. However, it is worth noting that the decreasing trend of pressure drop becomes negligible when the filtration efficiency of PAN membranes is 95–96%. Furthermore, a drastic increase of pressure drop is observed when the mean fiber diameter is lower than 60 nm. This unusual phenomenon could be due to the largely decreased pore size (Supplementary Fig. S6), which can significantly influence the airflow distribution around the periphery of fibers.

Plotting the variation curve of equilibrium factors of various PAN membranes versus their fiber diameters revealed that the drag force on the periphery of fibers also changed significantly with decrease of pore size (as shown in Fig. 6c), indicating the vital role of pores in the pressure drop changes. This scaling law also indicated that the slip-effect will gradually weaken and ultimately disappear with decreasing distance of adjacent fibers. Therefore, the relationship between pore size and pressure drop change is a prerequisite to determine the effective range of slip-effect. As illustrated in Fig. 6d, the variation of pressure drop showed a trend of gradual decrease with the decrease of pore size (Supplementary Fig. S6). Finally, the pressure drop showed no change at all when the pore size was reduced to less than 3.5 μm, indicating that the distance of adjacent fibers greatly influences the slip-effect caused by the change of air flow state. This interesting trend is also important for the structural design and preparation of electrospun membranes with slip-effect. Based on these results, the PAN-8-M3 membrane would be evaluated more thoroughly in the following study.

### PM_2.5_ purification performance

**Figure 7:**
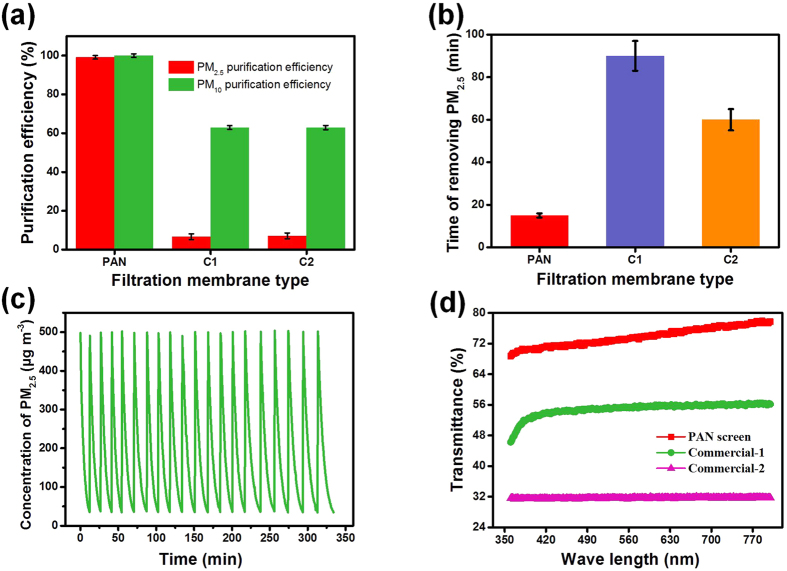
Purification properties, long term service property, and light transmittance of PAN-8-M3 fibrous membranes. (**a**) Purification efficiency of PM_2.5_ and PM_10_, (**b**) Time of removing PM_2.5_ from 500 to 35 μg cm^−3^, (**c**) The long term recycling operational performance, and (**d**) Transmittance in the range of visible light wavelength of PAN, commercial-1, and commercial-2.

Due to their slip-effect and appropriate pore size, the PAN-8-M3 fibrous membranes were used to evaluate the PM_2.5_ purification properties. As shown in Fig. 7a, the slip-effect functional PAN-8-M3 fibrous membranes exhibited very high PM_2.5_ purification efficiency of 99.09% and PM_10_ purification efficiency of 99.98%, indicating that the pore structure developed by random stacking of nanofibers could effectively separate the ultrafine particulate matter from the airstream through the capturing mechanisms of interception, diffusion and inertial impaction^19^. More importantly, the PAN-8-M3 fibrous membranes exhibited superior purification efficiency of 99.28% towards particulate matter (130–150 μg m^−3^) in the real environment of Shanghai, and also displayed stable filtration efficiency and pressure drop (see details in Supplementary Fig. S7, Supplementary Discussion and Supplementary Methods). In addition, two commercial state-of-the-art window screens were tested for comparison. Commercial-1 and commercial-2 samples exhibited much lower PM_2.5_ purification efficiencies of 6.60% and 7.10%, respectively, and PM_10_ purification efficiencies of 62.90% and 68.92%, respectively. The lower purification efficiencies of these commercial materials could be due to their relatively large pore size (see details in Supplementary Fig. S8), resulting in attenuation of the physical interception and inertial impaction^42^.

To demonstrate the combined benefits of low pressure drop and high PM_2.5_ purification efficiency of PAN fibrous membranes, the clean air delivery rate was measured. This parameter is defined by the time taken for the PM_2.5_ concentration to decrease from 500 to 35 μg m^−3^. As illustrated in Fig. 7b, it took the PAN-8-M3 filter membrane only 15 min to achieve this. This result demonstrates that even highly polluted air could be purified in a relatively short time due to low pressure drop of 40 Pa and high PM_2.5_ removal efficiency of 99.09%. Furthermore, the two commercial window screens (C1 and C2) were also tested for comparison. C1 and C2 took much longer times of 67 min and 37 min, respectively, to produce clean air. Their relatively poor performance is likely due to the lower purification efficiency of C1 (6.6%) causing difficulty in removing PM_2.5_ and the high pressure drop of 136 Pa for C2 leading to decrease in air capacity handled in unit time.

The deposition of particles containing inorganic and organic components would have considerable effect on the PM purification performance of fibrous membranes. Therefore, the long term recycling operational performance of slip-effect structured PAN-8-M3 fibrous membranes was investigated under severe contamination conditions, in which the PM_2.5_ index was equivalent to or greater than 500. As shown in Fig. 7c, the PAN fibrous membrane maintained a clean air delivery rate of 15 min without any change, even after testing for 20 cycles. This behavior could be attributed to (a) the physical interception manner of the fibrous membrane, which would not cause reduction of filtration efficiency like electric materials^43^, and (b) the strong adhesive force between nanofibers and particles due to the robust dipole–dipole and induced-dipole intermolecular forces, which would prevent removal of the adhered particles. This result is consistent with other studies^44^. Besides PM_2.5_ purification performance, light transmittance was also evaluated, as it is another important parameter for an air filter with low pressure drop in practical application. As illustrated in Fig. 7d, the window screen fabricated by PAN-8-M3 membranes showed the optimal transmittance of 77%, while the other commercial window screens C1 and C2 showed only 50% and 35% optimal transmittance, respectively, indicating that the slip-effect structured PAN-8-M3 membranes have great potential to be used in window screens, which require high capacity of light transmittance. In addition, we also explored the feasibility of scaling-up of the electrospinning process for the as-prepared air filters. The requirements for large-scale production of the air filters have been discussed in the Supplementary Discussion section.

## Discussion

The successful fabrication of slip-effect functional nanofibrous membranes provides an effective platform for designing and constructing nanofibrous materials for air filtration with low air resistance.

In summary, we have successfully fabricated novel slip-effect functional nanofibrous membranes with decreased air resistance due to the slip flow of air molecules on the periphery of nanofibers, by controlling the diameter of single fibers and the aperture size of fiber assembly. These PAN-based fibrous materials with optimized pore size were developed as a model system for proof-of-concept. The fiber diameter range of 60–100 nm (86% of fibers) was found to be the most effective range for the slip-effect, as it is similar to the mean free path length of air molecules (65.3 nm). This allows the air molecules to bypass the nanofibers with maximum probability, therefore decreasing the pressure drop. Significantly, equilibrium factor *τ* = *d*_*f*_/*d*^2^ was introduced to establish the relationship between the structure of membranes (fiber diameter and pore size) and drag force of airflow on the periphery of fibers in order to understand the influence of the distance of adjacent fibers on airflow behavior. Furthermore, the most effective range of aperture size (>3.5 μm) for slip-effect was determined. Finally, the PAN fibrous membranes with the optimized parameters showed ultralow air resistance of 29.5 Pa, high PM_2.5_ purification efficiency of 99.09%, good transmittance of 77% and long service life. We envision that these novel slip-effect structured nanofibrous membranes would enable further development of improved filters such as ultralow resistance respirators, air purifiers and transparent window screens which can be used for individual protection, clean rooms, and indoor air purification. Furthermore, the efficient structural design of these new electrospun nanofibrous membranes (multilayered structure and cavity structure) would pave the way for new types of slip-effect functional nanofibrous materials for use in various applications.

## Methods

### Materials

Polyacrylonitrile (PAN) (*M*_*w*_ = 51000, density = 1.184 g cm^−3^) was obtained from J&K Scientific Co. Ltd., China. *N,N*-Dimethylformamide (DMF) was purchased from Shanghai Macklin Biochemical Co. Ltd., China. Lithium chloride (LiCl) was supplied by TCI (Shanghai) Development Co. Ltd., China. The hot-rolled non-woven substrate (polyethylene terephthalate) for the collection of nanofibers possessed negligible filtration efficiency (2%) and pressure drop (0 Pa) when the airflow velocity equaled to 5.3 cm s^−1^, and was kindly supplied by Hainan Xinlong Nonwoven Co. Ltd., China.

### Preparation of polymer solutions

Typically, five bottles of 9 wt% precursor solutions were prepared by dissolving PAN powder into LiCl/DMF liquid with vigorous stirring at 25 °C for 12 h. The corresponding concentrations of LiCl were 0, 0.004, 0.008, 0.012, and 0.016 wt%, respectively. A conductivity tester (OKD-656, Shenzhen OK instrument technology, China) was used to measure the conductivity of polymer solutions with various concentrations of LiCl. The solution viscosity was measured using a rotary viscometer (NDJ-4, Shanghai Ni Run intelligent technology Co. Ltd., China). Surface tension was measured using a tensiometer (Sigma 703D, DKSH, China). The temperature and relative humidity in the test chamber were 23 ± 2 °C and 45 ± 3%, respectively.

### Fabrication of slip-effect functional PAN membranes

The PAN nanofibrous membranes were obtained by employing a DXES-4 electrospinning device (Shanghai Oriental Flying Nanotechnology Co. Ltd., China). Typically, the PAN polymer solution was drawn into plastic syringes, which were then clamped to a supporting frame capable of moving right and left continuously. Then, the homogeneous solution was extruded through 5 G metal needle with a controllable infusion velocity of 0.3 mL h^−1^. Simultaneously, the DC voltage of 30 kV was applied at the tips of the needles, leading to the formation of a stable jet stream. In addition, the temperature and relative humidity in the electrospinning chamber were 23 ± 2 °C and 45 ± 3%, respectively. The obtained ultrafine PAN fibrous membranes were assembled on an earthed metallic tumbling barrel covered by a nonwoven substrate, which rotated at a speed of 50 rpm, and the tip-to-collector distance was 15 cm. The resultant PAN nanofibrous membranes were soaked in isopropyl alcohol (IPA) for 2 min and then dried for 20 min at the temperature of 80 °C in a vacuum oven to neutralize the electrostatic force. The obtained membranes fabricated from the polymer solution with various LiCl concentrations of 0, 0.004, 0.008, 0.012, and 0.016 wt% were denoted as PAN-0, PAN-4, PAN-8, PAN-12, and PAN-16, respectively. In addition, the fibrous membranes with various levels of filtration efficiencies (44–45, 66–68, 86–88, and 95–96%) were fabricated by controlling the electrospinning time (see details in Supplementary Methods) and the corresponding fibrous membranes were denoted as PAN-M1, PAN-M2, PAN-M3, and PAN-M4. The PAN-8 membranes with the filtration efficiency level of 87–88% were denoted as PAN-8-M3.

### Characterization

Scanning electron microscope (SEM, TM 3000, Hitachi Ltd., Japan) was used to analyze the morphology of PAN fibrous membranes. Fiber diameters of the membranes were measured using an image analyzer (Adobe Photoshop CS 6). An automatic filtration performance tester purchased from Huada Instrument and Equipment Co. Ltd., China was employed to evaluate the filtration performance. The charge neutralized sodium chloride (NaCl) monodisperse aerosol particles possessing mass-average particle size of 0.3–0.5 μm and geometric standard less than 1.86, were generated using an atomizing air pump and then passed through the test samples with a valid test area of 100 cm^2^. The NaCl aerosol particles were detected by a laser airborne particle counter at ambient temperature (25 ± 2 °C) and humidity (45 ± 5%). The pressure drops of samples were measured by a flow gauge and two electronic pressure transmitters. All the pressure drop tests were conducted under a continuous airflow fixed at 5.3 cm s^−1^. In addition, if the structure of the material is known, then the obtained values of pressure drop for the samples are constant. The detailed information about the test equipment for evaluating the filtration properties is given in Supporting Information Fig. S9. The aperture sizes of fabricated materials were measured employing a capillary flow porometer based on Laplace’s equation (CFP-1100AI, Porous Materials Inc., USA). Porosity of the fabricated membrane was obtained employing the following formula:


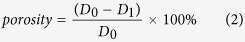


where *D*_0_ represents the density of raw PAN and *D*_1_ signifies the density of fibrous membranes. A UV-VIS-NIR Spectrophotometer (U-4100, HITACHI, Japan) was used to measure the transmittance.

### PM purification efficiency measurement

With regard to PM_2.5_ purification efficiency measurement, simulated particles were generated by the burning of a cigarette. The generated white smoke particles possessed a broad granulometric distribution from <0.3 μm to >10 μm, with a large proportion of particulate matter being <1 μm. The concentration of particulate matter at the entrance was adjusted by diluting the smoke particulate matter with clean air to a severe pollution level, in which the PM_2.5_ concentration was greater than or equivalent to 500 μg m^−3^. The concentration of particulate matter in the airtight test cabin was detected with and without filters by a PM_2.5_ professional concentration detection instrument (SDL 301, Nova Fitness, China) and the PM_2.5_ purification efficiency was obtained by calculating the concentration difference before and after filtration. The clean air delivery rate and long term recycling operational performance were measured in an airtight cabin, which contained a small air purification machine with the airflow of 14 L min^−1^ and two PM_2.5_ professional concentration detection instruments (SDL 301, Nova Fitness, China) (see details in Supplementary Fig. S10). The filter materials were placed in the air inlet and sealed with double-sided adhesive tape to guarantee the air passing into the air purification machine through filter materials completely. The clean air delivery rate was obtained by calculating the time taken for the PM_2.5_ concentration to decrease from 500 to 35 μg m^−3^. The long term recycling operational performance was evaluated by testing the PM_2.5_ concentration decrease from 500 to 35 μg m^−3^ for 20 cycles.

## Additional Information

**How to cite this article**: Zhao, X. *et al*. Slip-Effect Functional Air Filter for Efficient Purification of PM_2.5_. *Sci. Rep.* **6**, 35472; doi: 10.1038/srep35472 (2016).

## Supplementary Material

Supplementary Information
