# Supplementary material for: Slip-Effect Functional Air Filter for Efficient Purification of PM2.5
Source: Sci Rep. 2016 Oct 17;6:35472. doi: 10.1038/srep35472 (PMC5066256; doi:10.1038/srep35472)
Supplement: Supplementary Information [file srep35472-s1.pdf]

Supplementary Information for

Slip-Effect Functional Air Filter for Efficient Purification of $PM_{2.5}$

Xinglei Zhao[1], Shan Wang[1], Xia Yin[1], Jianyong Yu[2] & Bin Ding* [1,2]

[1] Key Laboratory of Textile Science & Technology, Ministry of Education, College of Textiles, Donghua University, Shanghai 201620, China

[2] Nanofibers Research Center, Modern Textile Institute, Donghua University, Shanghai 200051, China

* Corresponding author: Prof. Bin Ding, E-mail address: binding@dhu.edu.cn

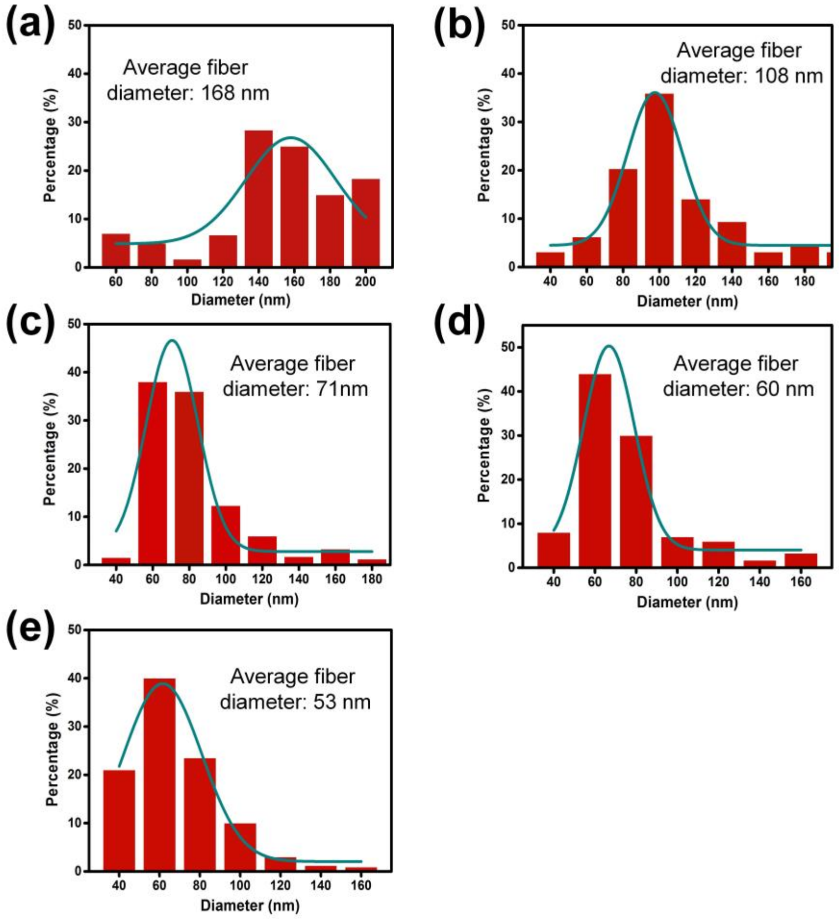

Figure S1 │ The distribution of fiber diameter of PAN fibrous membranes. Histogram showing the distribution of fiber diameter of PAN fibrous membranes fabricated from polymer solution containing various concentrations of LiCl: (a) 0, (b) 0.004, (c) 0.008, (d) 0.012, and (e) 0.016 wt%.

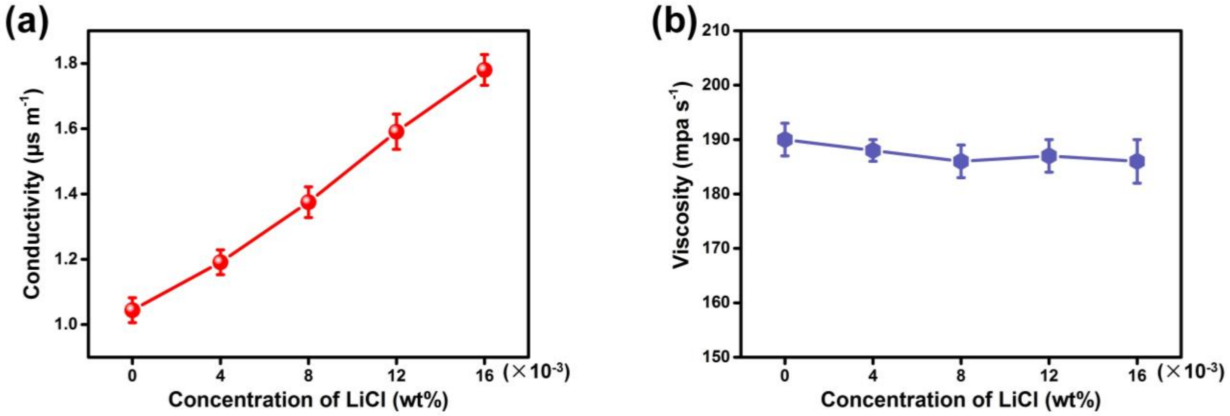

Figure S2 │ Solution properties of PAN polymer solutions. (a) Conductivity and (b) viscosity of PAN polymer solutions with various concentrations of LiCl.

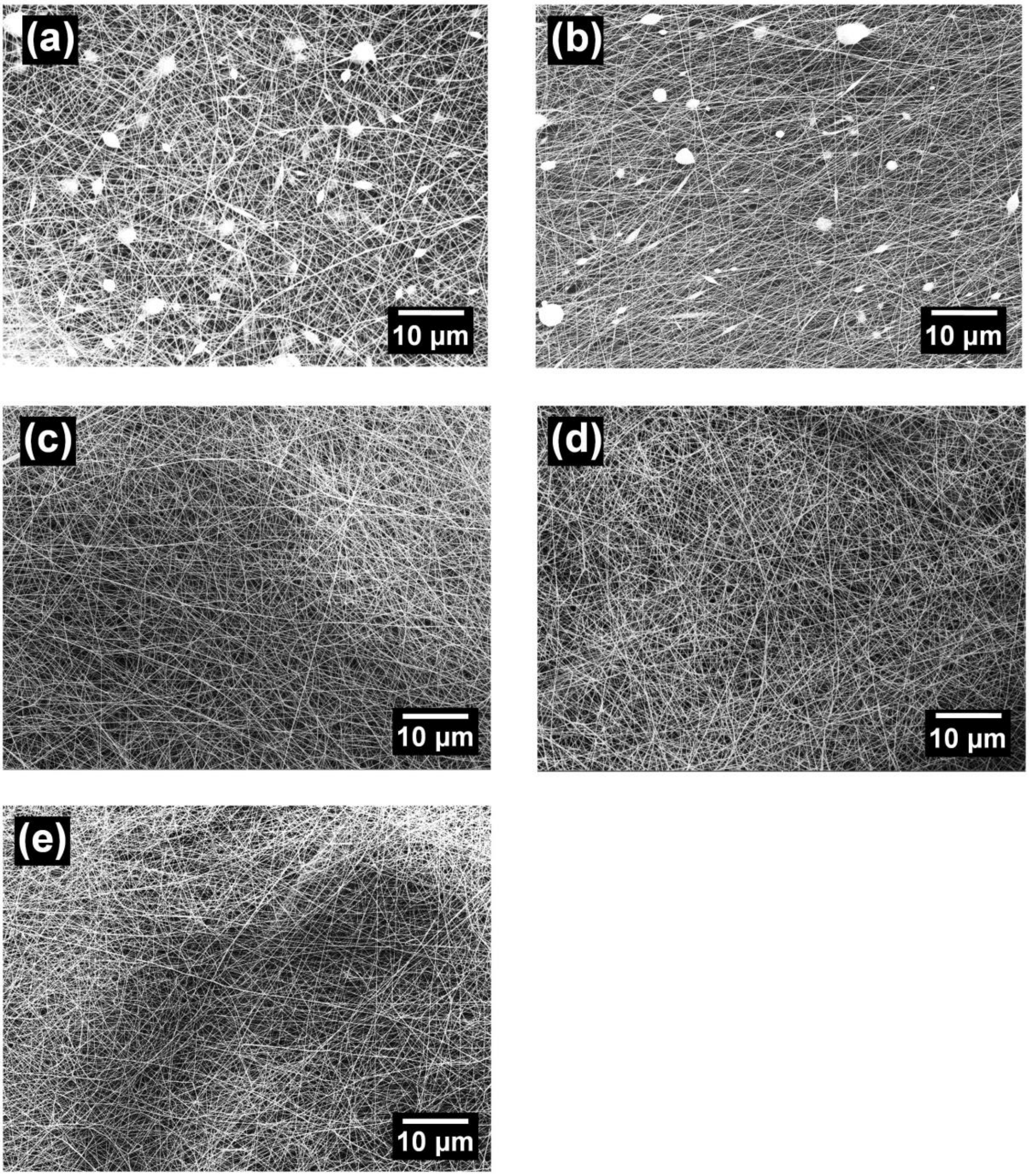

Figure S3 │ The SEM images of PAN fibrous membranes. The SEM images of PAN fibrous membranes fabricated from polymer solutions containing various concentrations of LiCl: (a) 0, (b) 0.004 wt%, (c) 0.008 wt%, (d) 0.012 wt%, and (e) 0.016 wt%.

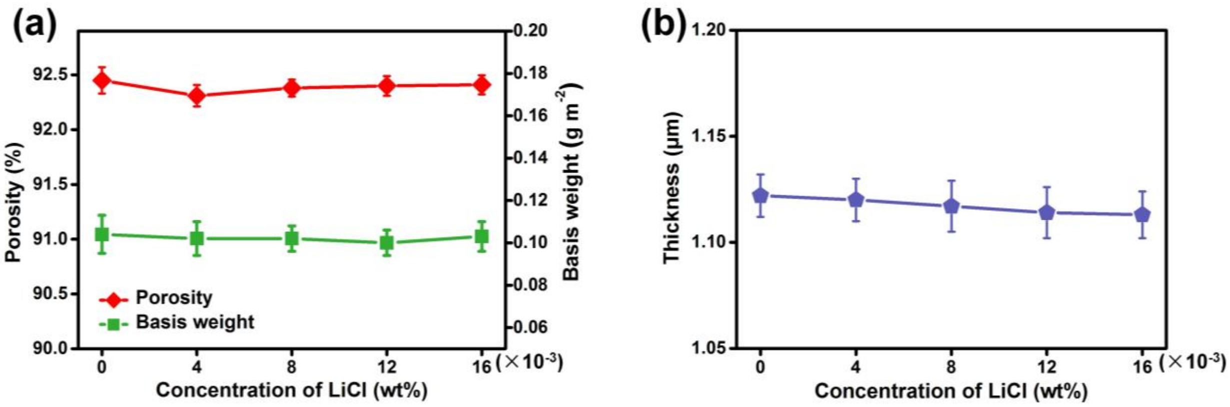

Figure S4 | Structural properties of PAN fibrous membranes. (a) Porosity and basis weight, and (b) thickness of PAN fibrous membranes fabricated from polymer solutions containing various concentrations of LiCl.

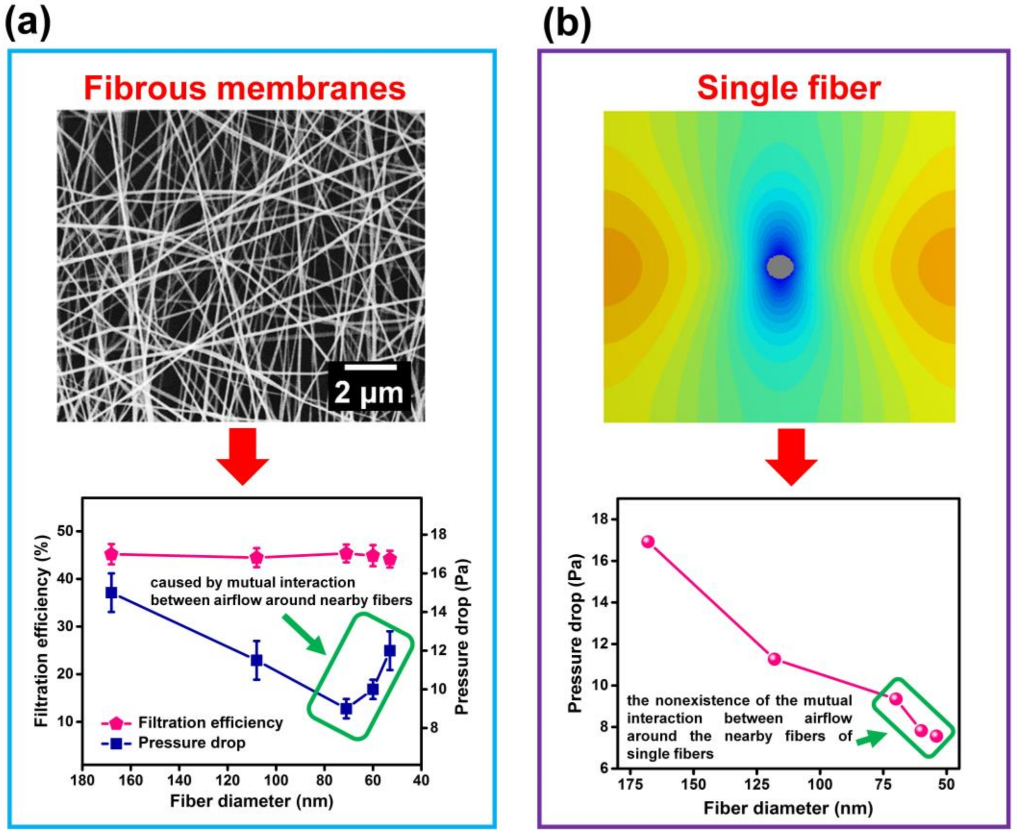

Figure S5 | Explanation for the difference in the pressure drop trends between fibrous membranes and single fiber. (a) The explanation for the difference in the pressure drop trends of fibrous membranes. (b) The explanation for the difference in the pressure drop trends of single fiber.

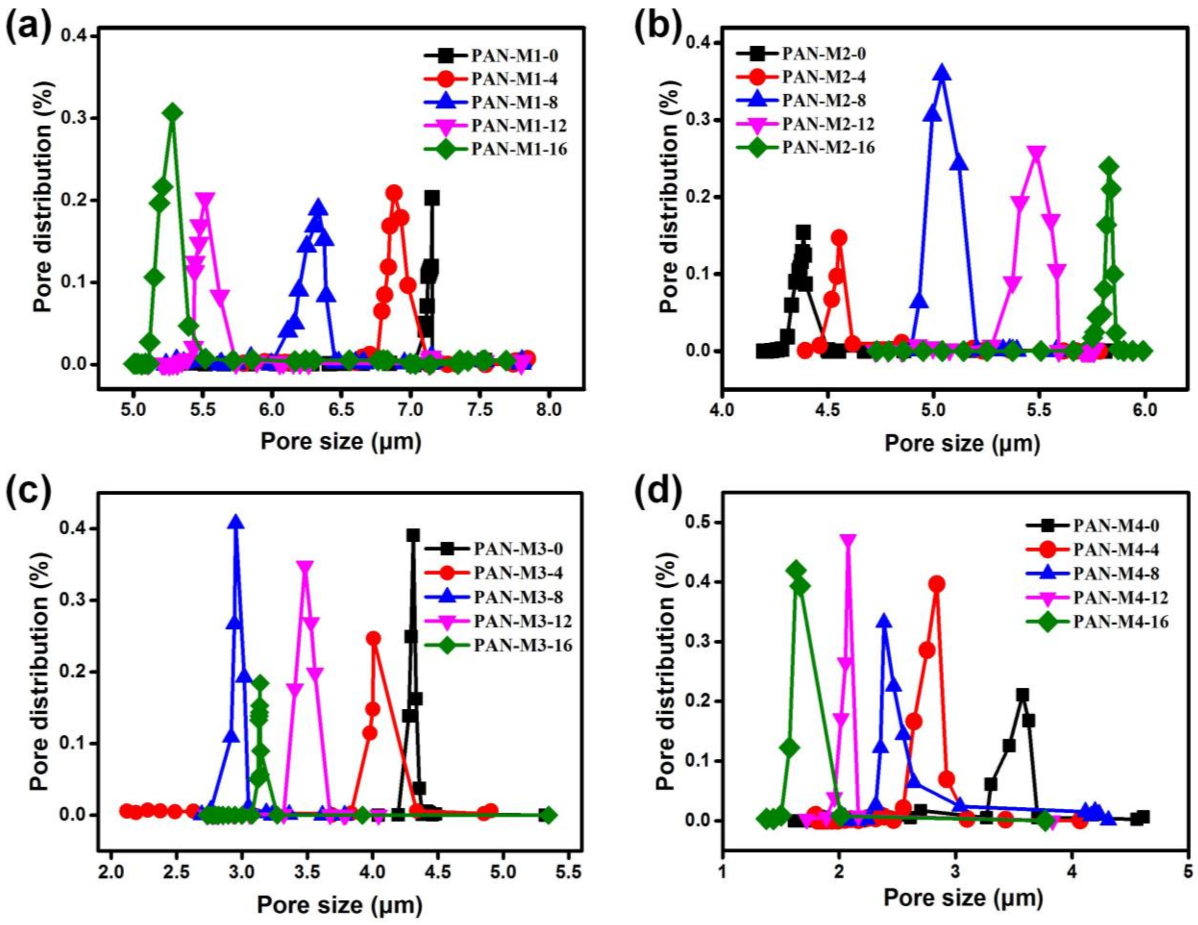

Figure S6 │ Pore distribution of PAN fibrous membranes with various levels of filtration efficiencies. (a) The pore size of PAN-M1. (b) The pore size of PAN-M2. (c) The pore size of PAN-M3. (4) The pore size of PAN-M4.

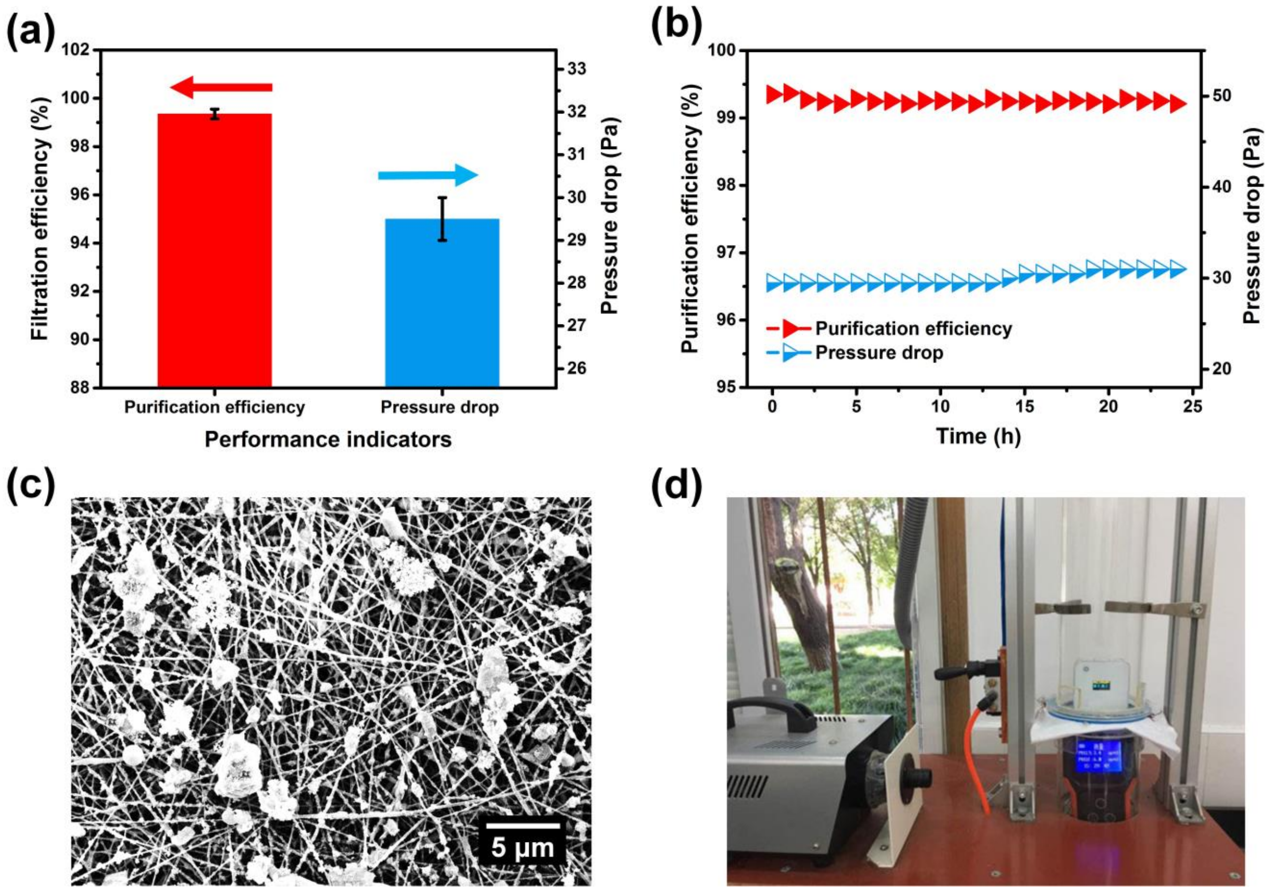

Figure S7 │ Field test (Shanghai) performance and long-term performance of PAN-8-M3 fibrous membranes. (a) The initial purification efficiency towards real particulate matter and pressure drop of PAN-8-M3 fibrous membranes. (b) The long-term purification efficiency towards real particulate matter and pressure drop of PAN-8-M3 fibrous membranes. (c) SEM showing the morphology of PAN-8-M3 air filter after 24 h particulate matter capture test. (d) Experimental setup for evaluating property of filter media using real particulate matter.

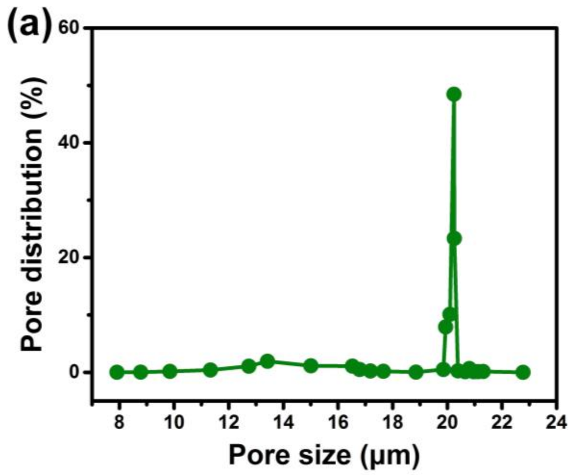

Figure S8 │ Pore size distribution of commercial materials. The pore size distribution of (a) commercial-1 and (b) commercial-2.

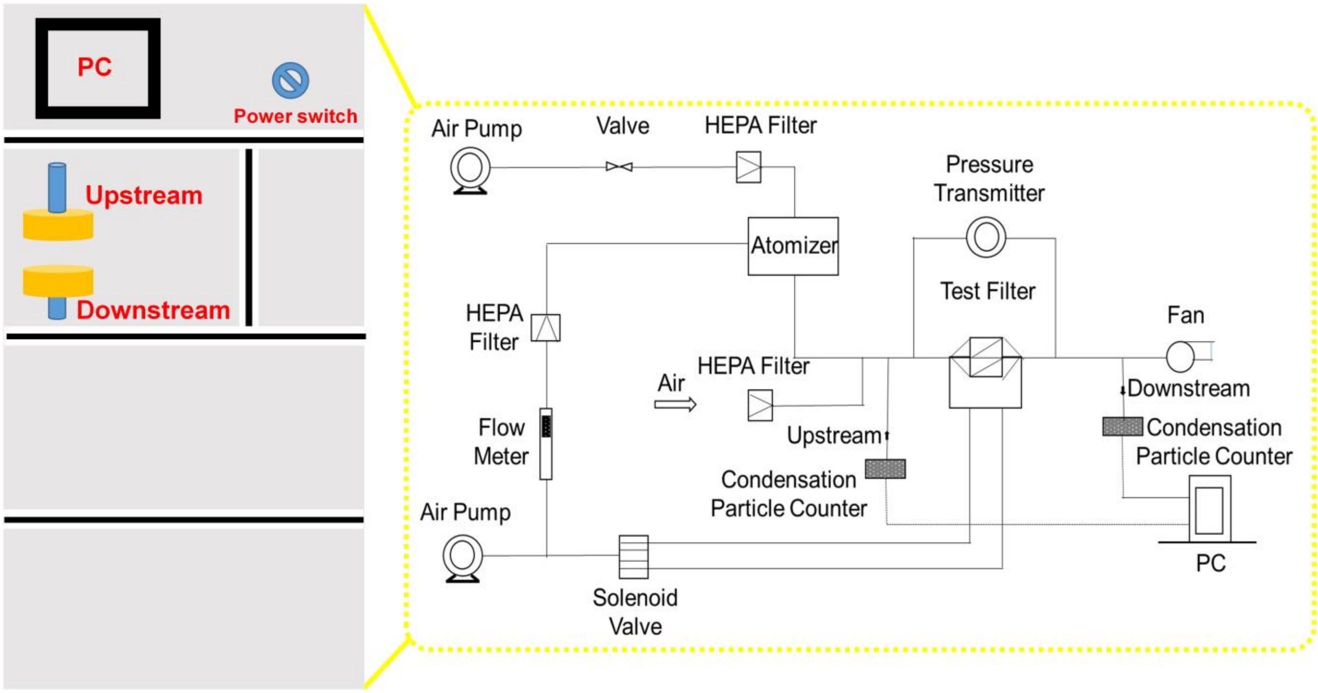

Figure S9│Experimental setup for evaluating the properties of filter media.

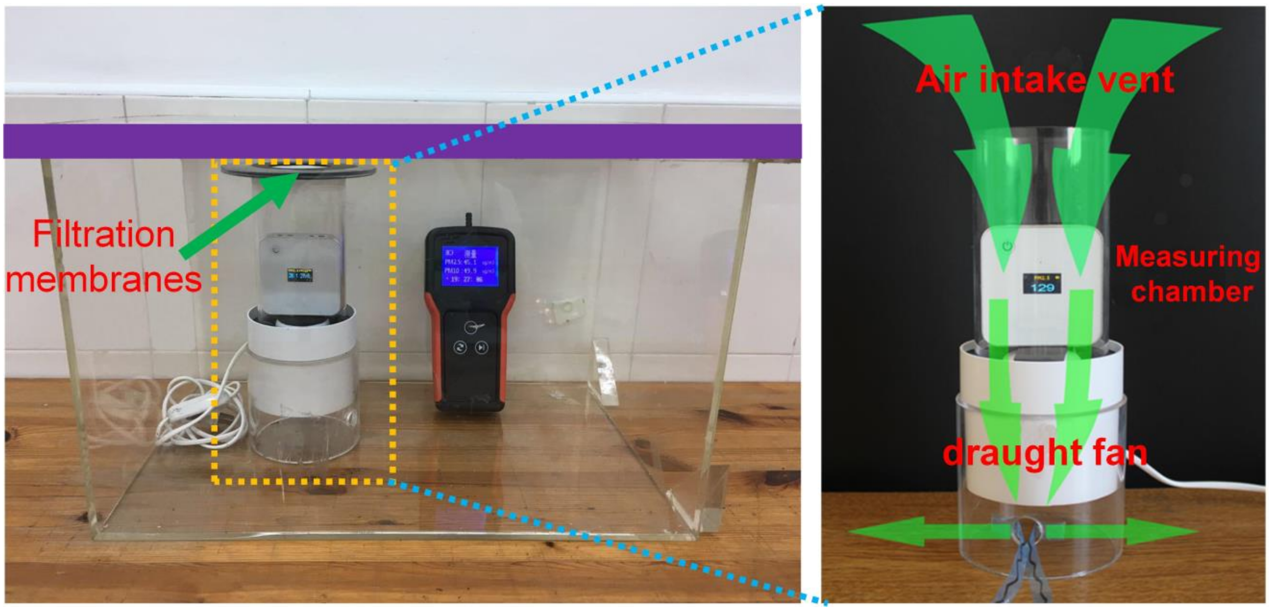

Figure S10 | Equipment for purification efficiency measurement of simulated $PM_{2.5}$

Supplementary Discussion

Performance of the slip effect functional air filters in a field test. Testing the purification performance using the particulate matter in real environments can indicate the feasibility of pratical application for these fibrous membranes. Thus, we investigated the purification efficiency of PAN-8-M3 fibrous membranes towards particulate matter (130-150 μg m^{-3}) in the real environment of Shanghai, China. As shown in Figure S6a, the purification efficiency for real particulate matter was 99.28%, which was higher than the purification efficiency for NaCl aerosol particles. This phenomenon could be due to the larger size of real particulate matter than that of NaCl aerosol particles (300-500 nm) (as shown in Fig. S6c). In addition, the long-term performance of the PAN-8-M3 fibrous membranes was evaluated. After 15 h, the PAN-8-M3 filter still exhibited stable $PM_{2.5}$ purification efficiency of 99.23% towards particulate matter with only a slight increase in the pressure drop (by 2 Pa), indicating their stable long-term performance.

Scaling-up of the electrospinning process for the as-prepared air filter. Having demonstrated the excellent purification performance of the new membranes, it is important to assess whether they can be produced efficiently on a large scale. We can realize the large-scale preparation from the following four aspects. First, the number of nozzles should be increased to improve the production speed and the needles would be configured reasonably to ensure the uniformity of the products. Second, uniform temperature and humidity conditions in the electrospinning region would be guaranteed to ensure uniformity of the product. Third, a covering layer would be added onto the nanofibrous layer to prevent their breakage due to lower mechanical strength. Fourth, suitable apparatus for testing the air permeability and thickness of fibrous membranes would be installed to ensure quality control.

Supplementary Methods

The experimental details on preparing PAN-M1 to PAN-M4. To obtain fibrous membranes with various levels of filtration efficiencies using PAN polymer solutions containing various LiCl concentrations of 0, 0.004, 0.008, 0.012, and 0.016 wt%, the electrospinning time is a critical factor. For PAN-M1, the electrospinning times of PAN-M1-0, PAN-M1-4, PAN-M1-8, PAN-M1-12, and PAN-M1-16 fibrous membranes were 5 min 55 s, 5 min 40 s, 5 min 30 s, 5 min 25 s, and 5 min 10 s, respectively. For PAN-M2, the electrospinning times of PAN-M2-0, PAN-M2-4, PAN-M2-8, PAN-M2-12, and PAN-M2-16 fibrous membranes were 10 min, 9 min 45 s, 9 min 30 s, 9 min 25 s, and 9 min 10 s, respectively. For PAN-M3, the electrospinning times of PAN-M3-0, PAN-M3-4, PAN-M3-8, PAN-M3-12, and PAN-M3-16 fibrous membranes were 16 min 15 s, 16 min 05 s, 15 min 55 s, 15 min 45 s, and 15min 30 s, respectively. For PAN-M4, the electrospinning times of PAN-M4-0, PAN-M4-4, PAN-M4-8, PAN-M4-12, and PAN-M4-16 fibrous membranes were 27 min, 26 min 45 s, 26 min 30 s, 26 min 15 s, and 25 min 55 s, respectively. In addition, all the technical parameters of electrospinning were consistent

(voltage of 30 kV, distance of 15 cm, the temperature of 23 ± 2 °C and the relative humidity of 45 ± 3%).

The regulation of pore size for investigating the effect of them on slip effect. The regulation of pore size of PAN fibrous membranes fabricated from polymer solutions containing various LiCl concentration of 0, 0.004, 0.008, 0.012, and 0.016 wt% were controlled through regulating the electrospinning time and keeping the technical parameters of electrospinning (voltage of 30 kV, distance of 15 cm, the temperature of 23 ± 2 °C and the relative humidity of 45 ± 3%) consistent. Taking the fabrication of PAN-M1 fibrous membranes as an example, to ensure the filtration efficiency with nearly identical values, the electrospinning times of PAN-M1-0, PAN-M1-4, PAN-M1-8, PAN-M1-12, and PAN-M1-16 fibrous membranes were 5 min 55 s, 5 min 40 s, 5 min 30 s, 5 min 25 s, and 5 min 10 s, respectively. After the fabrication, we measured the pressure drop and pore size of corresponding fibrous membranes. Ultimately, combining the test result of pressure drop and pore size, the relationship between the pressure drop and pore size of fibrous membranes was established and then the most effective range of aperture size (> 3μm) for slip-effect was determined.

Real particles purification efficiency measurement: With regard to purification efficiency measurement of real particular matter, particles were obtained from the real environments of Shanghai. A vacuum pump was used to inhale the outside air into the test chamber with the airflow of 14 L min^{-1}. The number concentration of particular matter in the airtight test cabin was detected with and without filters by a $PM_{2.5}$ professional concentration detection instrument (SDL 301, Nova Fitness) and the $PM_{2.5}$ purification efficiency was obtained by calculating the concentration difference before and after filtration.
